# M1-Polarized Macrophages Promote Self-Renewing Phenotype of Hepatic Progenitor Cells with Jagged1-Notch Signalling Involved: Relevance in Primary Sclerosing Cholangitis

**DOI:** 10.1155/2018/4807145

**Published:** 2018-12-24

**Authors:** Heli Li, Shiran Sun, Qing Lei, Ping Lei, Xiong Cai, Chidan Wan, Guanxin Shen

**Affiliations:** ^1^Department of Hepatobiliary Surgery, Union Hospital, Tongji Medical College, Huazhong University of Science and Technology, Wuhan 430022, China; ^2^Department of Immunology, Tongji Medical College, Huazhong University of Science and Technology, Wuhan 430030, China

## Abstract

The immunologic interaction between parenchyma cells and encircling inflammatory cells is thought to be the most important mechanism of biliary damage and repair in primary sclerosing cholangitis (PSC). Monocytes/macrophages as master regulators of hepatic inflammation have been demonstrated to contribute to PSC pathogenesis. Macrophages coordinate with liver regeneration, and multiple phenotypes have been identified with diverse expressions of surface proteins and cytokine productions. We analyzed the expression of Notch ligand Jagged1 in polarized macrophages and investigated the relevance of Notch signalling activation in liver regeneration. M1 or M2 macrophages were generated from mouse bone marrow-derived macrophages (BMDMs) by classical or alternative activation, respectively. Then, the expression levels of Jagged1 (*Jag1*) of each phenotype were measured. The effects of polarized BMDMs on the expression of hepatic progenitor cell- (HPC-) specific markers and hairy and enhancer of split-1 (HES1) in HPCs in coculture were also analyzed. Monocyte-macrophage and Notch signalling-associated gene signatures were evaluated in the GEO database (access ID: GSE61260) by gene set enrichment analysis (GSEA). M1 macrophages were found associated with elevated Jag1 expression, which increased the fraction of HPC with self-renewing phenotypes (CD326^+^CD44^+^ or CD324^+^CD44^+^) and HES1 expression level in cocultured HPC. Blocking Jagged1 by siRNA or antibody in the coculture system attenuates HPC self-renewing phenotypes as well as HES1 expression in HPC. GSEA data show that macrophage activation and Notch signalling-associated gene signatures are enriched in PSC patients. These findings suggest that M1 macrophages promote an HPC self-renewing phenotype which is associated with Notch signalling activation within HPC. In the liver of PSC patients, the prevalence of activated macrophages, with M1 polarized accounting for the main part, is associated with increment of Notch signalling and enhancement of HPC self-renewal.

## 1. Introduction

Primary sclerosing cholangitis (PSC) is a life-long liver disease characterized by chronic cholangitis associated with destruction of intra- and extrahepatic bile ducts. Dynamic cholangiocyte damage eventually results in cholangiocyte death, regeneration, and fibrosis-cirrhosis. Though the etiology and pathogenic mechanisms currently warrant further elucidation, the gene expression profiles of PSC are regarded as sharing traits with both inflammatory bowel disease and autoimmune diseases. Convincing human leukocyte antigen (HLA) gene correlations, in association with gene signatures representing T-cell function, supports the implication of acquired immunity in PSC pathogenesis and categorizes PSC as an autoimmune disease [1]. The immunologic interaction between hepatic parenchyma cells and encircling inflammatory cells is thought to be the most important mechanism underlying biliary damage and repair [2].

Progenitor/stem cells are cells with a capacity for self-renewal and differentiation to regenerate adult tissues. In several organs, the lineages of stem cells, fate committed progenitor cells, and terminal differentiated cells are well documented. The adult liver is known to have outstanding regenerative capability, while in many chronic liver diseases, e.g., chronic viral hepatitis, alcohol-related liver disease, nonalcoholic fatty liver disease (NAFLD), and PSC/primary biliary cholangitis (PBC)/autoimmune hepatitis [3], efficient regeneration from hepatocyte/cholangiocyte replication becomes impaired. In this setting, unique epithelium populations known as hepatic progenitor cells (HPC) activate and expand. HPC has long been regarded to reside in the Canals of Hering, which locates at the terminal branches of the biliary tree, typically exhibiting intermediate phenotypes between hepatocellular and biliary lineages as characterized and sorted by morphology and cell surface marker profiles (e.g., CD13, CD44, CD324, CD326, and Dlk1), and they are thought to be committed bi-potential progenitor cells [4].

It has been proposed that HPC may be differentially activated in diverse inflammatory milieus. Thus, several inflammatory cytokines, such as interleukin-6 (IL-6), tumour necrosis factor-alpha (TNF-*α*), and interferon-gamma (IFN-*γ*), have been identified to stimulate HPC expansion. In addition, several studies focusing on illuminating the molecular frameworks for HPC regulation mechanisms have corroborated a number of developmental associated signalling pathways (e.g., Wnt, Notch, HGF-c-MET, EGF, and FGF), playing crucial roles in regulating HPC self-renewal/proliferation and differentiation. Nevertheless, cellular events, especially signalling producing cells and subsequent cell-cell communications, in these complex regulatory mechanisms have not been well described [5].

Monocytes/macrophages are regarded as one of the principal regulators of hepatic regional immunity, regeneration, and fibrosis and are also reported to contribute to PSC pathogenesis [6]. Monocytes/macrophages are characterized by high diversity in function, transcriptional profiles, cell surface markers, and cytokine production [7]. The heterogeneous phenotype of macrophages attributes to discrete subpopulations that differentiate in response to signals associated with inflammatory milieus. The proinflammatory phenotype (M1) moderates the protection of microorganisms and contributes to inflammation-induced damage. Differential regulation of Notch receptors and autonomous activation of Notch signalling in M1-polarized macrophage have been reported and associated with cooperation between the Toll-like receptor (TLR) and Notch signalling pathways [8–10]. In the present study, we hypothesize that classical activated macrophages present Notch ligand Jagged1 to HPC in a cell-cell interaction manner, triggering Notch signalling in HPC and subsequently enhancing the HPC phenotype. Since several studies have highlighted several crucial functions of liver macrophages, which are chiefly exerted by macrophages derived from infiltrating monocytes, but not by resident Kupffer cells in the set of chronic hepatitis [11], in the current study, we applied a coculture assay using primary murine HPC and bone marrow-derived macrophages (BMDMs) to investigate the signal interaction between the two cell populations.

## 2. Materials and Methods

### 2.1. Mice

All experiments with mice were approved by the Experimental Animal Facility of Tongji Medical College and the Research Administration Office in Union Hospital affiliated to Tongji Medical College, Huazhong University of Science and Technology. Throughout the experiments, mice were maintained in a temperature- and humidity-controlled specific pathogen-free (SPF) environment. Wild-type female pregnant C57BL/6 mice and wild-type C57BL/6 mice, 6–8 weeks of age, weighing 20–30 g were purchased from HFK Bioscience (Beijing, China, SPF grade). EGFP transgenic C57BL/6 mice (three 6-week-old male and three 6-week-old female) were obtained from HFK Bioscience for maintenance and breeding.

### 2.2. Purification and Maintenance of Hepatic Progenitor Cells

Cell suspensions from fetal livers of ED13.5 C57BL/6 mice were prepared as described previously [12]. Purification of HPC from single-cell suspensions was carried out by utilizing the MACS® cell sorting system (Miltenyi, Auburn, CA) with the rat anti-mouse CD324, also known as E-cadherin, antibody (clone ECCD-1, Takara, Mountain View, CA, or Calbiochem, San Diego, CA) and goat anti-rat IgG microbeads. Purification was performed by the protocol recommended by the manufacturer in indirect labelling option. The positive selection of CD324^+^ cells was plated in a 6-well plate precoated with Matrigel® matrix (Corning, Corning, NY). The purity of sorted cells was accessed by flow cytometry using PE-conjugated rat anti-mouse CD324 antibody (clone 114420, R&D Systems, Minneapolis, MN).

For short-term cell culture or rapid expansion of cells, the complete culture medium was the basal medium containing 30 mg/l L-proline (Sigma-Aldrich, St. Louis, MO), 10^−7^ M dexamethasone (Sigma-Aldrich), 10 mM nicotinamide (Sigma-Aldrich), 1 mM ascorbic acid-2 phosphate (Sigma-Aldrich), and 1x penicillin-streptomycin (Invitrogen, Carlsbad, CA), supplemented with 5% FBS (Invitrogen), 20 ng/ml HGF (PeproTech, Rocky Hill, NJ), 20 ng/ml EGF (PeproTech), and 1x ITS-X (Invitrogen). For long-term cell maintenance or cell reprogram, the serum-free culture medium was basal medium containing 30 mg/l L-proline, 10^−7^ M dexamethasone, 10 mM nicotinamide, 1 mM ascorbic acid-2 phosphate, 0.05% BSA (Sigma-Aldrich), and 1x penicillin-streptomycin supplemented with 5 mM Y-27632 (Selleck, Houston, TX), 2.5 mM A-83-01 (Selleck), 15 mM CHIR99021 (Selleck), 10 ng/ml EGF, and 1x ITS-X. All plates (Corning) were precoated with 0.5% Matrigel (growth factor-reduced, Corning) dissolved in DMEM/F12 (Invitrogen) medium overnight at 4°C. The medium was replaced every 2 days. For cell passage, TrypLE (Invitrogen) is used for dissociating cells from surfaces.

### 2.3. In Vitro Induction of Bone Marrow-Derived Macrophages

Wild-type female C57BL/6 mice or EGFP transgenic C57BL/6 mice were used for induction of BMDMs. Briefly, mouse bone marrow cells were rinsed from cavitas of femurs and tibias with RPMI 1640 (Invitrogen). Bone marrow progenitor cells were seeded in 10 ml RPMI 1640 containing 100 U/ml M-CSF, 10% inactivated FBS, and 1x penicillin-streptomycin in 100 mm polystyrene tissue culture dishes (Corning). After three days of stimulation, 5 ml of new prepared medium was added. At day seven, BMDMs (>99% macrophages based on flow cytometry using parameter F4/80) were collected for the experiments. BMDMs were stimulated with lipopolysaccharide (LPS, 0.1 *μ*g/ml, Sigma-Aldrich) and interferon- (IFN-) *γ* (20 ng/ml, PeproTech) or with IL-4 (20 ng/ml, PeproTech) to induce polarization towards M1 or M2 phenotypes, respectively.

### 2.4. Coculture Assay

After seeding and differentiating BMDMs as described above, the starved hepatic progenitor cells were seeded in the same wells at a ratio 1 : 1, and the coculture system was maintained for 24 hours in an incubator.

### 2.5. Flow Cytometry and Cell Sorting

For flow cytometry analyses, after blockade with 10% normal goat serum (Invitrogen) at 37°C for 30 min, cells were incubated with fluorescence-labelled antibodies (Table 1) according to the manufacturer's recommendation. Fluorescence-labelled (Alexa Fluor 568) goat anti-rabbit secondary antibody was used for indirect labelling. Then, cells were tested with FACS LSR II (Becton-Dickinson, Franklin Lakes, NJ) applying appropriate negative, isotype, or fluorescence minus one (FMO) control. Data were analyzed using FlowJo software (Tree Star). In detail, after excluding debris, doublets, and dead cells by forward scattering and side scatter gating, positively labelled cell fractions were gated and calculated using an appropriate control to set a marker such that 0.1% to 2% cells fall to the right. To separate HPC from EGFP^+^ macrophages in a coculture system for further gene expression assay, cell suspensions were sorted with FACS Aria II (Becton-Dickinson) applying parameter GFP.

### 2.6. RNA Interference, Plasmid Transfection, and Jagged1 Blockage In Vitro

We have designed vectors for RNA interference targeting mouse *Jag1* (*siJag1*; targeting sequence 5-CTGGTGGAGGCCTGGGATTCC-3; GenBank accession number NM_013822.5). jetPRIME reagents (Polyplus-transfection®, Illkirch-Graffenstaden, France) were used following the manufacturer's protocols. Before M1 polarization, macrophages were transfected with *siJag1* or a control vector. CD339 (Jagged1) Functional Grade Monoclonal Antibody (10 *μ*g/ml, clone HMJ1-29, Invitrogen) was used to block Jagged1 in the coculture system as described [13].

### 2.7. RNA Extraction and Quantification

Cell total RNA was extracted with TRIzol® Reagent (Invitrogen), and cDNA was prepared using a RevertAid First Strand cDNA Synthesis Kit (Thermo Fisher Scientific, Baltics, UAB). Quantitative real-time polymerase chain reaction (qRT-PCR) analyses were performed using a SYBR Green Real-Time PCR Master Mix (Toyobo, Osaka, Japan) in a LightCycler® (Bio-Rad Laboratories, Hercules, CA). Specific oligonucleotides were synthetized according to the sequences shown in Table 2.

### 2.8. Protein Extraction and Western Blot Assay

Equal amounts of protein from macrophages or HPC were analyzed by 10% SDS-PAGE and blotted using a specific antibody as described in Table 1. Protein expression was quantified by means of optic density measured by ImageJ software (http://imagej.nih.gov/ij/). Data were normalized to *β*-actin.

### 2.9. Computational Analysis

The GSE61260 dataset, comprising 14 PSC and 38 healthy control liver tissues, was downloaded from the NCBI GEO (https://www.ncbi.nlm.nih.gov/geo/). The raw CEL files derived in the HuGene 1.1 ST gene array (Affymetrix, Santa Clara, CA) for GSE61260 were normalized. To identify gene sets enriched in either control or PSC patients, Gene Set Enrichment Analysis (GSEA) was applied as previously described [14].

### 2.10. Statistical Analyses

All statistical analyses were calculated by R software (http://www.r-project.org/). Mean values were compared using Student's *t*-test (two groups) or one-way ANOVA (three or more groups), A *p* value <0.05 was considered to be statistically significant. Results are represented by mean and standard deviation (SD). All qRT-PCR and flow cytometry data were collected from three independent experiments. The Western blots shown are representative images from three independent experiments.

## 3. Results

### 3.1. Successful Induction of BMDM Polarization towards M1 or M2 Phenotypes *In Vitro*

**Figure 1:**
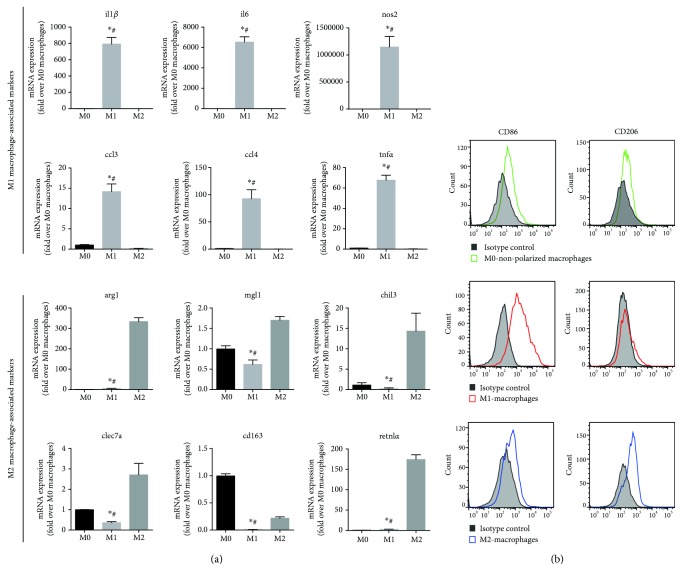
Induction of BMDM polarization towards M1 or M2 phenotype *in vitro*. (a) Quantitative analysis of M1 macrophage-associated marker (*Il1β*, *Il6*, *nos2*, *Tnfα*, *Ccl3*, and *Ccl4*) and M2 macrophage-associated marker (*Arg1*, *Mgl1*, *Chil3*, *Cd163*, *Clec7a*, and *Retnlα*) expression in M0, M1, and M2 macrophages. (b) Membranous expressions of CD86 and CD206 levels on M0, M1, and M2 macrophages were determined by flow cytometry. The histogram is presented. ^∗^*p* < 0.05 vs M0 macrophages, ^#^*p* < 0.05 vs M2 macrophages.

To assess the effect of polarized macrophages, BMDMs were either treated with LPS + IFN-*γ* to polarize towards the M1 phenotype or treated with IL-4 to polarize towards the M2 phenotype; cells administrated with the vehicle were regarded as M0 (nonpolarized) macrophages. Then, the mRNA expression of several M1 or M2 macrophage-specific genes was examined by qRT-PCR. As shown in Figure 1(a), the mRNA expressions of *il1β*, *il6*, *nos2*, *tnfα*, *ccl3*, and *ccl4* were significantly increased in M1 macrophages compared with the expression in M0 or M2 macrophages. After M2 polarization, a significant increase in the mRNA expression of *arg1*, *mgl1*, *chil3*, *clec7a*, and *retnlα* can be found in M2 macrophages (Figure 1(a)). Though *cd163* mRNA expression was downregulated after M2 polarization, *cd163* mRNA expression in M2 macrophages was significantly higher than in M1 macrophages (Figure 1(a)). By flow, M1 macrophages showing increased frequencies of CD86, but not CD206, exhibit a reverse phenotype of M2 macrophages, while M0 macrophages exhibit an increase in neither CD86 or CD206 expression (Figure 1(b)). These data indicate that we have successfully induced the classical activated (M1) macrophages as well as alternative activated (M2) macrophages *in vitro*.

### 3.2. M1 Macrophages Promote CD44^+^CD326^+^ Population of HPC in a Coculture System

**Figure 2:**
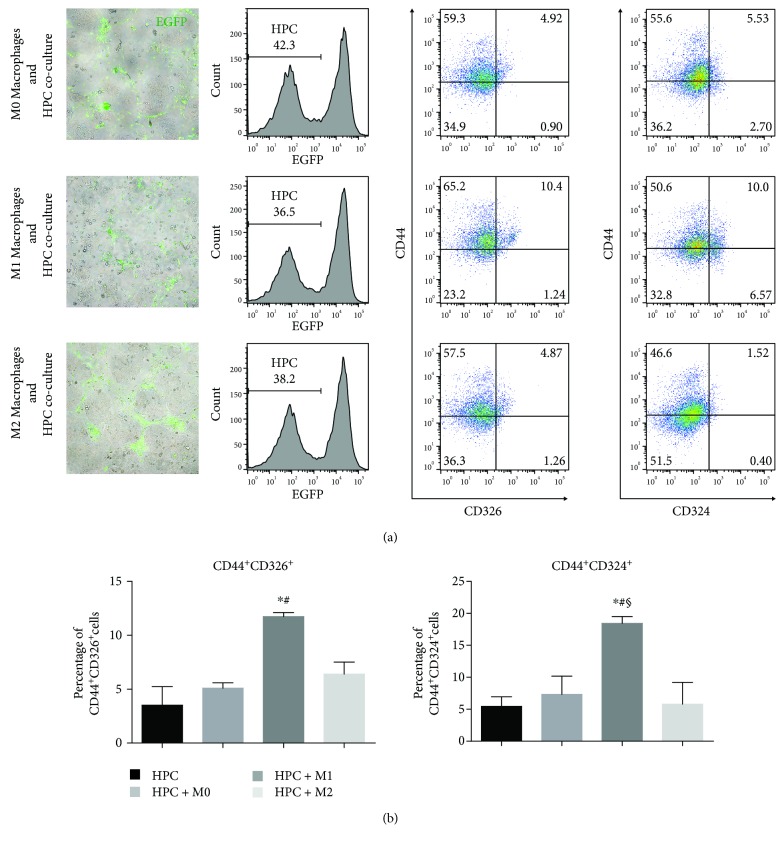
M1 macrophages promote a self-renewing phenotype of HPCs in a coculture system. (a) HPCs were cocultured (24 hours) with M0, M1, or M2 macrophages from EGFP transgenic C57BL/6 mice. After flow cytometry analysis, CD44^+^CD324^+^ and CD44^+^CD326^+^ cell fractions of HPC are highlighted by scatter plots. (b) Flow cytometry data were calculated and represented. ^∗^*p* < 0.05 vs HPC, ^#^*p* < 0.05 vs M0 macrophages cocultured with HPC, and ^§^*p* < 0.05 vs M2 macrophages cocultured with HPC.

Macrophages derived from infiltrating monocytes have many crucial functions in regulating hepatic regional inflammation, which is very important for HPC to get a promoted capacity of self-renewal or differentiation. To evaluate the diverse functions of polarized macrophages influencing HPC biology, we designed an in vitro coculture system containing HPC and polarized macrophages at the ratio of 1 : 1 for 24 hours. In the coculture assay, M1 macrophages significantly increased the cell population of CD44^+^CD326^+^ HPCs (Figures 2(a) and 2(b)). In contrast, HPC cocultured with M0 or M2 macrophages did not show a significant increase in the CD44^+^CD326^+^ population compared with HPC alone (Figures 2(a) and 2(b)). Similar results were obtained when identifying CD44^+^CD324^+^ population in HPCs within the coculture system. These data indicated that M1 macrophages can promote a self-renewing phenotype of HPC in our coculture system.

### 3.3. Elevation of Notch Ligand Jagged1 Expression in Classical Activated (M1) BMDMs Is Associated with Activation of Notch Signalling in HPC

**Figure 3:**
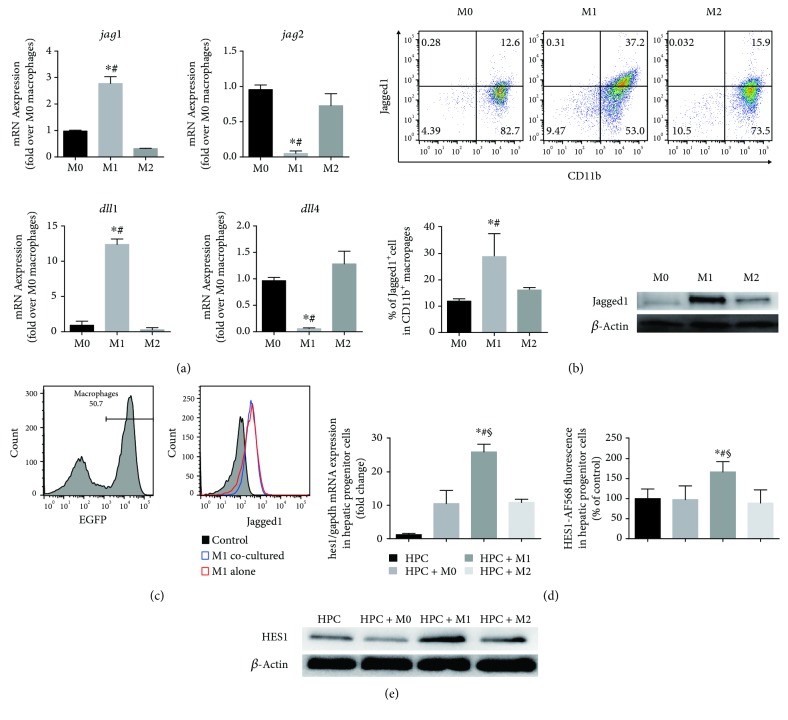
Classical macrophage activation is associated with elevated Notch ligand Jagged1 expression in macrophages and activation of Notch signalling in cocultured HPCs. (a) Quantitative analysis of Notch ligands (*Jag1*, *Jag2*, *Dll1*, and *Dll4*) expression in M0, M1, and M2 macrophages. (b) Jagged1 levels on M1 macrophages are confirmed by flow cytometry and Western blots. Flow cytometry results are represented by scatter plot, and calculated flow cytometry data are represented; ^∗^*p* < 0.05 vs M0 macrophages and ^#^*p* < 0.05 vs M2 macrophages. (c) Histograms from flow cytometry analysis represent Jagged1 expressions in M1 macrophages from EGFP transgenic C57BL/6 mice cultured with or without HPC for 24 hours. (d) Graphs show relative mRNA expression levels of HES1 *in* HPC alone or in HPC cocultured with M0, M1, or M2 macrophages; levels of HES1 staining (Alexa Flour 568) in HPC were determined by static cytometry. Graphs show calculated relative expression of HES1 in HPC cocultured with M0, M1, or M2 macrophages (percentage of HPC culture alone). ^∗^*p* < 0.05 vs HPC alone, ^#^*p* < 0.05 vs M0 macrophages cocultured with HPC, and ^§^*p* < 0.05 vs M2 macrophages cocultured with HPC. (c) Representative Western blots showing HES1 protein levels in HPC after coculturing with M0, M1, or M2 macrophages.

Further, we investigate the mRNA expression of Notch ligands in each polarized population by qRT-PCR. Expression of *Jag1* and DLL1 showed significant elevation in M1 macrophages, in comparison with other phenotypes of macrophages, while JAG2 and DLL4 expression levels were obviously decreased in M1 macrophages. These data are consistent with the previous study [10]. Flow cytometry and Western blot were applied to determine the protein level of JAG1 expression in polarized macrophages. We confirmed that M1 macrophage had an elevated level of Notch ligand Jagged1 expression. However, we found that *Jag1* mRNA expression levels did not correspond to levels of protein. As is well known, mRNA levels are often not reflected in protein levels since gene expression is controlled at multiple stages and various ways. We speculate that there might be a feedback loop within M2 macrophages controlling Jagged1 protein expression supported by findings that *Jag1* is a direct Notch target in specific cells. [15] To further exclude the interference of possible flexible *Jag1* expression within 24 hours of culture on coculture assay results, we gated the expression of JAG1 in M1 macrophages from EGFP transgenic C57BL/6 mice, with or without coculture, with HPC by flow cytometry. The result shows that JAG1 expression level stays stable during the 24-hour coculture period with HPC (Figure 3(c)).

Noting that a high level of JAG1 expression in M1 macrophages and JAG1 is closely associated with liver development and regeneration, we hypothesized that M1 macrophages might mediate the increase of HPC self-renewing phenotype by activating the Notch signalling pathway in HPC. After 24 hours of coculture, HPCs for extracting mRNA and protein were sorted from the cocultured cell mixture by parameter EGFP-negative. qRT-PCR, flow cytometry, and WB were employed to analyze the mRNA expression and protein level of HES1 in HPC after coculturing with macrophages. As shown, M1 macrophages exhibit the strongest HES1 expression levels in both mRNA and protein (Figures 3(d) and 3(e)). Hence, it is reasonable for us to conclude that M1 macrophages regulate the expression of HES1 in HPC by activating Notch signalling.

### 3.4. Jagged1 Suppression in the Coculture System Attenuates Self-Renewing Population as well as HES1 Expression in HPC

**Figure 4:**
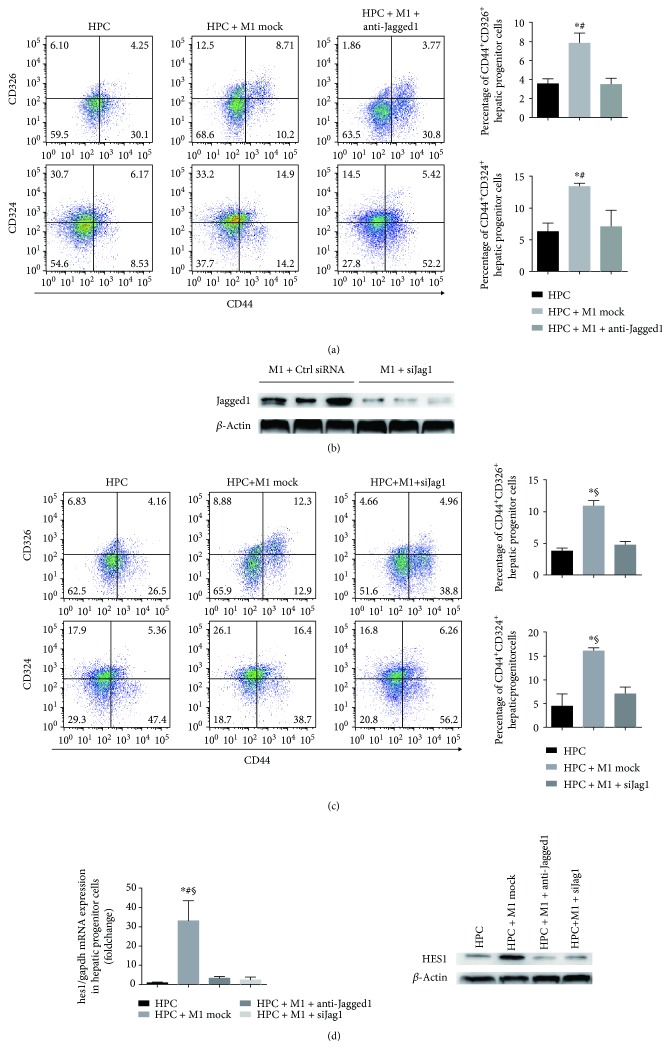
Blocking Jagged1 in a coculture system attenuates hepatic progenitor cell self-renewal as well as HES1 expression in HPC. (a) HPCs were cocultured (24 h) with M1 macrophages from EGFP transgenic C57BL/6 mice with or without the existence of anti-Jagged1 mAb (10 *μ*g/ml). Graphs show fluctuation of CD44^+^CD324^+^ and CD44^+^CD326^+^ cell fractions in HPC cultured alone, cultured with M1 macrophages or with anti-Jagged1 mAb in coculture system. (b) Representative Western blots showing protein levels of Jagged1 in M1 macrophages transfected with mock or *siJag1*. In the cases that macrophages were transfected with mock or *siJag1* vectors before M1 polarization, CD44^+^CD324^+^ and CD44^+^CD326^+^ cell fractions of HPC are highlighted by calculated histogram. (c) Graphs and representative Western blots showing protein levels of HES1 and mRNA expression of HES1 in HPC when cocultured with M1-*siJag1* or cocultured with M1 macrophages and 10 *μ*g/ml anti-Jagged1 mAb. ^∗^*p* < 0.05 vs HPC alone, ^#^*p* < 0.05 vs M1 macrophages cocultured with HPC + anti-Jagged1 antibody, and ^§^*p* < 0.05 vs M1 macrophages cocultured with HPC + *siJag1*.

To confirm that M1 macrophages regulate the cocultured HPC phenotype through Jagged1-Notch interaction, anti-Jagged1 mAb was added in coculture assays to block the ligand. In the presence of blocking mAb against Jagged1, the CD326^+^CD44^+^ or CD324^+^CD44^+^ population of HPC was downregulated when cocultured with M1 (Figure 4(a)). As an alternative strategy to manipulate Jagged1-initiated Notch signalling, we further utilized *siJag1* to knock down the expression of *Jag1* in M1 macrophages (Figure 4(b)). As shown in Figure 4(b), M1 macrophages transfected with the *siJag1* plasmid shows a downregulated expression of *Jag1*. Knockdown of *Jag1* in M1 macrophages attenuates CD44^+^CD326^+^ as well as CD44^+^CD324^+^ cell fractions within cocultured HPC. In addition, the mRNA expression and protein level of HES1 in M1 macrophages cocultured HPC are both inhibited by blocking Jagged1 with mAb or knocking down JAG1 in M1 macrophages (Figure 4(c)). Collectively, our data suggest that M1 macrophages initiated Jagged1-Notch interaction active Notch signalling within HPC. Notch signalling activation in HPC is associated with enhanced HPC self-renewing phenotype.

### 3.5. Monocyte-Macrophage and Notch Signalling Pathway Gene Signatures Are Enriched in Primary Sclerosing Cholangitis Liver Samples

**Figure 5:**
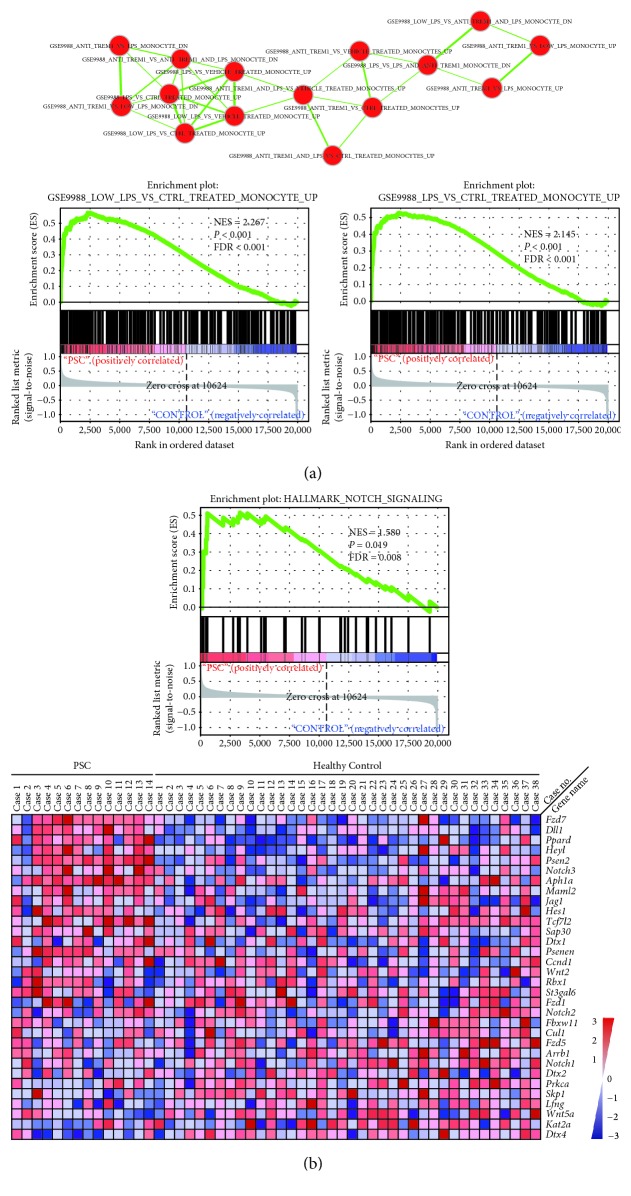
Genome-wide transcriptomic analysis of primary sclerosing cholangitis and control. Monocyte-macrophage and Notch signalling pathway gene signatures are enriched in primary sclerosing cholangitis liver samples. (a) Cytoscape and Enrichment maps were used for the visualization of monocyte-macrophage-associated GSEA results. Nodes represent enriched gene sets, grouped and annotated by similarity according to the related gene sets. Node size is proportional to the total number of genes within the gene set. NES: normalized enrichment score; FDR: false discovery rate. (b) GSEA-generated heat map of core enrichment genes in hallmarks of the Notch signalling pathway upregulated in PSC.

To identify the functional signatures of monocyte-macrophage and Notch signalling pathway-associated genes that were differentially enriched in PSC patients and normal control, GSEA was conducted in the GEO database (access ID: GSE61260). We screened gene enrichment among 4872 immunologic signature-related gene sets in the Molecular Signatures Database (MSigDB), and we found that multiple monocyte-macrophage-associated gene sets were enriched in PSC patients (data not shown). Especially, a group of gene sets derived from gene expression data of previous study (Access ID: GSE9988) [16], in which human monocytes received the administration with untreated, anti-TREM-1 mAb, or LPS, were enriched in PSC patients vs. healthy control. As shown in Figure 5(a), gene sets representing an upregulated gene signature in monocytes after LPS treatment or low-dose LPS treatment vs. monocytes were among the top-scoring gene enrichment sets. The results may suggest that monocyte/macrophage activation-associated indexes are most correlated with PSC patients vs. healthy control. Since LPS stimulation mostly induces classical activation of macrophages, it is also reasonable for us to speculate that M1 macrophage plays an important role in PSC pathogenesis. Then, we screened gene enrichment among 50 gene sets representing hallmark gene sets in MSigDB. We found that the Notch signalling pathway-associated gene signature was enriched in PSC patients (Figure 5(b)). Moreover, *Jag1* and HES1 were among the core enrichment genes (Figure 5(b)). Collectively, activated Notch signalling induced by Jagged1 is correlated with PSC patients vs. healthy control. Activated Notch signalling cannot be specifically located to HPC sites either in our experiment or in GSEA; thus, a detailed mechanism is far from being demonstrated yet. However, we still believe that Notch signalling is one of the most important regulators of HPC closely correlated with PSC pathogenesis or development.

## 4. Discussion

The present cell coculture assay demonstrates that bone marrow-derived M1 macrophages, but not M2 macrophages, enhance an HPC self-renewing phenotype in a direct coculture system. The mechanism can be interpreted by elevated expression of Jagged1 along with classical differentiation in BMDMs stimulating Notch signalling in cocultured HPC. Our findings support a possible cellular event featuring involvement of BMDM initiating Notch signalling in the regulation of HPC biology. In the liver of PSC patients, the disease status is associated with monocyte-macrophage activation (possibly favouring classical activation) and the Notch signalling-associated gene signature, possibly suggesting that M1 macrophages play a role in activating Notch signalling.

Continuous exposure to many pathogens makes the liver a unique organ. Several immune cells including monocyte-macrophages are actively involved in multiple hepatic immunological processes. Macrophage is the leading player of innate immunity with irreplaceable immunological functions, including phagocytosis, antigen presentation, and cytokine production. Cells of the monocyte-macrophage lineage serve a crucial role in the induction of chronic liver injury. Briefly, chemotaxis of bone marrow-derived Ly-6C^hi^ monocytes is enhanced by local CCL2 production, directing the cells to move to the liver. After settling down, Ly-6C^hi^ monocytes develop into infiltrating Ly-6C^+^ macrophages with the proinflammatory phenotype [17]. The infiltrating BMDMs trigger chronic liver injury and fibrosis by a mechanism involving transforming growth factor beta (TGF-*β*)/platelet-derived growth factor- (PDGF-) mediated hepatic stellate cell (HSC) activation [18]. The benefit of BMDM therapy has been demonstrated in murine models of developed liver fibrosis through a multifactorial mechanism of recruitment of anti-inflammatory host effector cells, anti-fibrosis, and proregeneration [19]. Of note, the proregenerative role of BMDM delivery has been associated with HPC activation [20]. This is in line with our coculture experiment data suggesting that BMDMs can maintain cocultured HPC in an undifferentiated phenotype.

There is an increasing acceptation that the monocyte-macrophage lineage may impact the HPC status in multiple pathways. It has been demonstrated that BMDMs express TWEAK activating HPCs [20]. More regulation roles have been defined in the process of cell fate determination. It has been suggested that macrophages expressing Wnt3a determine HPC fate differentiating towards hepatocyte by activating Wnt-*β*-catenin signalling [21], and then oncostatin M (OSM) excreted by macrophage stimulate the maturation of hepatocyte-committed cells through signal transducer and activator of transcription 3 (STAT3) signalling [22]. The polyfunctionality of macrophages in regulating HPC self-renewal and differentiation seemingly can be interpreted by the broad spectrum of macrophage phenotypes in various pathological conditions. Though dichotomous classification cannot fully mirror the complex biology of macrophage subpopulations in vivo, M1/M2 phenotypes may act as a simplified theoretical framework describing marked macrophage heterogeneity. The M1 and M2 status represents the phenotypic and functional extremes by not ontogenically defined subsets [23]. By coculturing M1 or M2 macrophages with HPCs, we observed a differential HPC self-renewing phenotype of two ends of the phenotypic spectrum. Our finding supports the idea that multiple subsets of macrophages modulate HPC status separately.

The Notch signalling pathway plays crucial roles in cell proliferation, differentiation, and survival in multiple cell types. It has been found that macrophage differentiation depends on the transcriptional regulator of Notch signalling [24]. Under the regulation of cooperative TLR and Notch signalling, autoamplification of Notch signalling mediated by Jagged1-RBP-J axis contributes to macrophage reciprocal regulation in the phenotype [8]. Consistent with the finding, we identified elevated *Jag1* gene expression in classically activated BMDMs. Since M1 macrophages have been reported to be able to activate Notch signalling in cocultured epithelial cells [10], we presume that macrophage-expressing Jagged1 is a Notch signalling trigger within neighbour cells but not limited to the macrophage itself. In our coculture system, inhibition of Jagged1-Notch interaction attenuates HPC self-renewing phenotype as well as HES1 expression. Notch signalling in regulating HPC biology has been extensively studied while its crucial roles are still in controversy. Though a large majority of studies believe that Notch signalling governs biliary differentiation of HPC [21, 25–27], contradictory results seem solid too [28, 29]. Diverse and even contrary biological effects of Notch signalling are believed to be attributed to its high specification varying in time, gene dose, and distinct cell type. Moreover, technically, most of the studies artificially modify Notch component gene expression, whose level will never be reproduced in any physiological or pathological conditions, or utilize broad inhibitors of Notch activation, complicating the issue [30]. In the current study, we do not test the differentiation capacity of HPC modulated by polarized macrophages, since fully hepatocellular or biliary differentiation in vitro is a complex process with multisteps and multisignals involved [31]. Further in vivo investigation will be interesting and straightforward.

Although we believe that Jagged1 produced by bone marrow-derived M1 macrophages is a crucial factor that mediates Notch activation that promotes the self-renewal-associated phenotype of HPC, we do not rule out the possibility that other signalling events produced by macrophages may also contribute to maintaining HPC self-renewal. In fact, a milestone study by Forbes et al. has demonstrated macrophages secreting Wnt3a to induce HPC-hepatocyte differentiation, while expression of Jagged1 in myofibroblasts activates Notch signalling within HPCs and hence their biliary differentiation [21]. Recently, it has been elucidated that macrophage-secreted TNF-*α* can lead to chromosomal instability in HPCs and promote the self-renewal of HPCs [32]. Given the complexity of HPC biology, we believe that multiple signalling pathways corporate to modulate HPC self-renewal and differentiation. Other important cellular and signalling events remain to be elucidated.

In conclusion, our findings suggest that M1 macrophages promote HPC self-renewing phenotype which is associated with activation of the Notch signalling pathway within HPC. In the liver of PSC patients, the prevalence of monocyte-macrophage is associated with increment of Notch signalling and enhanced HPC “stemness” phenotype. Regarding clinical translation, defining a specific phenotype and function of macrophages may help increase the predictability of effect when applying bone marrow-derived cell therapy in PSC patients. Further understanding of the involvement of HPC biology and Notch signalling in PSC pathogenesis may permit HPC and/or Notch signalling targeted therapy in the future.

## Figures and Tables

**Table 1:** Antibodies used for fluorescence studies and Western blot analysis.

Antibodies	Species	Reference	Antibody dilution
PE-conjugated anti-mouse F4/80	Rat mAb	BD Biosciences Catalog No. 565410	1 : 100
APC-Cy7-conjugated anti- mouse CD11b	Rat mAb	BD Biosciences Catalog No. 557657	1 : 100
V450-conjugated anti-Mouse CD44	Rat mAb	BD Biosciences Catalog No. 560451	1 : 100
PE/Cy7-conjugated anti-mouse/human CD324 (E-cadherin)	Rat mAb	BioLegend Catalog No. 147310	1 : 100
PE-conjugated anti-mouse CD324 (E-Cadherin)	Rat mAb	R&D Catalog No. FAB7481P	1 : 100
PE-conjugated anti-mouse CD339 (Jagged1)	Hamster mAb	BioLegend Catalog No. 130908	1 : 100
APC-conjugated anti-mouse CD326 (EpCAM)	Rat mAb	eBioscience Catalog No. 17-5791-80	1 : 100
APC-conjugated anti-mouse CD86 (B7-2)	Rat mAb	eBioscience Catalog No. 17-0862-82	1 : 100
FITC-conjugated anti-mouse CD206 (MMR)	Rat mAb	BioLegend Catalog No. 141703	1 : 100
Anti-mouse/human/rat Jagged1	Rabbit mAb	Invitrogen Catalog No. MA5-15012	1 : 500
Anti-mouse/human/rat/bovine HES1	Rabbit pAb	Invitrogen Catalog No. PA5-23283	1 : 500
Anti-mouse/human/rat/hamster *β*-actin	Rabbit pAb	Invitrogen Catalog No. PA1-46296	1 : 1000
Alexa Fluor 568-conjugated anti-rabbit IgG H&L	Goat pAb	Abcam Catalog No. ab175471	1 : 2000

**Table 2:** Primer sequences for quantitative real-time polymerase chain reaction.

Mouse gene	Sense	Antisense
*Jag1*	CCTGTCCATGCAGAACG	AGGCGAAACTGAAAGGC
*Jag2*	CAGATCCGAGTACGCTGTG	GGCTTCTTTGCATTCTTTGC
*Dll1*	GATACACACAGCAAACGTGACACC	TCCATCTTACACCTCAGTCGCTA
*Dll4*	CGAATGCCCCCCCAACT	GTTCGGCTTGGACCTCTGTTC
*Hes1*	CTCCCGGCATTCCAAGCTAG	AGCGGGTCACCTCGTTCATG
*Il1β*	GCAACTGTTCCTGAACTCAACT	ATCTTTTGGGGTCCGTCAACT
*Il6*	TGTGCAATGGCAATTCTGAT	GGTACTCCAGAAGACCAGAGGA
*Nos2*	GCAGCTGGGCTGTACAAA	AGCGTTTCGGGATCTGAAT
*Tnfα*	TGCCTATGTCTCAGCCTCTTC	GGTCTGGGCCATAGAACTGA
*Ccl3*	ATGAAGGTCTCCACCACTGC	CCCAGGTCTCTTTGGAGTCA
*Ccl4*	CAAACCTAACCCCGAGCAACA	GGTCTCATAGTAATCCATCACAAAGC
*Arg1*	GTGAAGAACCCACGGTCTGT	CTGGTTGTCAGGGGAGTGTT
*Retnlα*	TCCCAGTGAATACTGATGAGA	CCACTCTGGATCTCCCAAGA
*Mgl1*	TCTCTGAAAGTGGATGTGGAGG	GGAGGTGTAGGTGAAAGTCTCT
*Clec7a*	TCAAACATCGTCTCACCG	GTTGGGGAAGAATGCTG
*Chil3*	CATGAGCAAGACTTGCGTGAC	GGTCCAAACTTCCATCCTCCA
*Cd163*	TGGGTGGGGAAAGCATAACT	AAGTTGTCGTCACACACCGT
*Gapdh*	TCTCCACACCTATGGTGCAA	CAAGAAACAGGGGAGCTGAG

## Data Availability

The data used to support the findings of this study are available from the corresponding author upon request.

## References

[1] Jiang X., Karlsen T. H. (2017). Genetics of primary sclerosing cholangitis and pathophysiological implications. *Nature Reviews Gastroenterology & Hepatology*.

[2] Chung B. K., Karlsen T. H., Folseraas T. (2018). Cholangiocytes in the pathogenesis of primary sclerosing cholangitis and development of cholangiocarcinoma. *Biochimica et Biophysica Acta (BBA) - Molecular Basis of Disease*.

[3] Carpino G., Cardinale V., Folseraas T. (2018). Hepatic stem/progenitor cell activation differs between primary sclerosing and primary biliary cholangitis. *The American Journal of Pathology*.

[4] Boulter L., Lu W. Y., Forbes S. J. (2013). Differentiation of progenitors in the liver: a matter of local choice. *The Journal of Clinical Investigation*.

[5] Kitade M., Kaji K., Yoshiji H. (2016). Relationship between hepatic progenitor cell-mediated liver regeneration and non-parenchymal cells. *Hepatology Research*.

[6] Guicciardi M. E., Trussoni C. E., Krishnan A. (2018). Macrophages contribute to the pathogenesis of sclerosing cholangitis in mice. *Journal of Hepatology*.

[7] Murray P. J., Allen J. E., Biswas S. K. (2014). Macrophage activation and polarization: nomenclature and experimental guidelines. *Immunity*.

[8] Foldi J., Chung A. Y., Xu H. (2010). Autoamplification of Notch signaling in macrophages by TLR-induced and RBP-J-dependent induction of Jagged1. *Journal of Immunology*.

[9] Nomaguchi K., Suzu S., Yamada M., Hayasawa H., Motoyoshi K. (2001). Expression of Jagged1 gene in macrophages and its regulation by hematopoietic growth factors. *Experimental Hematology*.

[10] Ortiz-Masiá D., Cosín-Roger J., Calatayud S. (2016). M1 macrophages activate Notch signalling in epithelial cells: relevance in Crohn’s disease. *Journal of Crohn's & Colitis*.

[11] Karlmark K. R., Weiskirchen R., Zimmermann H. W. (2009). Hepatic recruitment of the inflammatory Gr1^+^ monocyte subset upon liver injury promotes hepatic fibrosis. *Hepatology*.

[12] Nitou M., Sugiyama Y., Ishikawa K., Shiojiri N. (2002). Purification of fetal mouse hepatoblasts by magnetic beads coated with monoclonal anti-E-cadherin antibodies and their in vitro culture. *Experimental Cell Research*.

[13] Sekine C., Moriyama Y., Koyanagi A. (2009). Differential regulation of splenic CD8^−^ dendritic cells and marginal zone B cells by Notch ligands. *International Immunology*.

[14] Subramanian A., Tamayo P., Mootha V. K. (2005). Gene set enrichment analysis: a knowledge-based approach for interpreting genome-wide expression profiles. *Proceedings of the National Academy of Sciences of the United States of America*.

[15] Manderfield L. J., High F. A., Engleka K. A. (2012). Notch activation of Jagged1 contributes to the assembly of the arterial wall. *Circulation*.

[16] Dower K., Ellis D. K., Saraf K., Jelinsky S. A., Lin L. L. (2008). Innate immune responses to TREM-1 activation: overlap, divergence, and positive and negative cross-talk with bacterial lipopolysaccharide. *The Journal of Immunology*.

[17] Ramachandran P., Pellicoro A., Vernon M. A. (2012). Differential Ly-6C expression identifies the recruited macrophage phenotype, which orchestrates the regression of murine liver fibrosis. *Proceedings of the National Academy of Sciences of the United States of America*.

[18] Tacke F., Zimmermann H. W. (2014). Macrophage heterogeneity in liver injury and fibrosis. *Journal of Hepatology*.

[19] Thomas J. A., Pope C., Wojtacha D. (2011). Macrophage therapy for murine liver fibrosis recruits host effector cells improving fibrosis, regeneration, and function. *Hepatology*.

[20] Bird T. G., Lu W. Y., Boulter L. (2013). Bone marrow injection stimulates hepatic ductular reactions in the absence of injury via macrophage-mediated TWEAK signaling. *Proceedings of the National Academy of Sciences of the United States of America*.

[21] Boulter L., Govaere O., Bird T. G. (2012). Macrophage-derived Wnt opposes Notch signaling to specify hepatic progenitor cell fate in chronic liver disease. *Nature Medicine*.

[22] Kamiya A., Kinoshita T., Ito Y. (1999). Fetal liver development requires a paracrine action of oncostatin M through the gp130 signal transducer. *The EMBO Journal*.

[23] Italiani P., Boraschi D. (2014). From monocytes to M1/M2 macrophages: phenotypical vs. functional differentiation. *Frontiers in Immunology*.

[24] Franklin R. A., Liao W., Sarkar A. (2014). The cellular and molecular origin of tumor-associated macrophages. *Science*.

[25] Spee B., Carpino G., Schotanus B. A. (2010). Characterisation of the liver progenitor cell niche in liver diseases: potential involvement of Wnt and Notch signalling. *Gut*.

[26] Kitade M., Factor V. M., Andersen J. B. (2013). Specific fate decisions in adult hepatic progenitor cells driven by MET and EGFR signaling. *Genes & Development*.

[27] Jeliazkova P., Jörs S., Lee M. (2013). Canonical Notch2 signaling determines biliary cell fates of embryonic hepatoblasts and adult hepatocytes independent of Hes1. *Hepatology*.

[28] Ortica S., Tarantino N., Aulner N., Israel A., Gupta-Rossi N. (2014). The 4 Notch receptors play distinct and antagonistic roles in the proliferation and hepatocytic differentiation of liver progenitors. *The FASEB Journal*.

[29] Gil-Garcia B., Baladron V. (2016). The complex role of NOTCH receptors and their ligands in the development of hepatoblastoma, cholangiocarcinoma and hepatocellular carcinoma. *Biology of the Cell*.

[30] Huntzicker E. G., Hötzel K., Choy L. (2015). Differential effects of targeting Notch receptors in a mouse model of liver cancer. *Hepatology*.

[31] Tanimizu N., Miyajima A., Mostov K. E. (2007). Liver progenitor cells develop cholangiocyte-type epithelial polarity in three-dimensional culture. *Molecular Biology of the Cell*.

[32] Li X. F., Chen C., Xiang D. M. (2017). Chronic inflammation-elicited liver progenitor cell conversion to liver cancer stem cell with clinical significance. *Hepatology*.

